# Crystal structure of *Trypanosoma cruzi* heme peroxidase and characterization of its substrate specificity and compound I intermediate

**DOI:** 10.1016/j.jbc.2022.102204

**Published:** 2022-06-27

**Authors:** Samuel L. Freeman, Vera Skafar, Hanna Kwon, Alistair J. Fielding, Peter C.E. Moody, Alejandra Martínez, Federico M. Issoglio, Lucas Inchausti, Pablo Smircich, Ari Zeida, Lucía Piacenza, Rafael Radi, Emma L. Raven

**Affiliations:** 1 School of Chemistry, University of Bristol, Bristol, United Kingdom; 2 Departamento de Bioquímica, Facultad of Medicina, Universidad de la República, Montevideo, Uruguay; 3 Centro de Investigaciones Biomédicas (CEINBIO), Facultad de Medicina, Universidad de la República, Montevideo, Uruguay; 4 Department of Molecular and Cell Biology and Leicester Institute of Structural and Chemical Biology, University of Leicester, Leicester, United Kingdom; 5 Centre for Natural Products Discovery, School of Pharmacy and Biomolecular Sciences, Liverpool John Moore University, Liverpool, United Kingdom; 6 CONICET-Universidad de Buenos Aires, Instituto de Química Biológica de la Facultad de Ciencias Exactas y Naturales (IQUIBICEN), Buenos Aires, Argentina; 7 Instituto de Tecnologia Química e Biológica António Xavier, Universidade Nova de Lisboa (ITQB NOVA), Oeiras, Portugal; 8 Laboratorio de Bioinformática, Departamento de Genómica, Instituto de Investigaciones Biológicas Clemente Estable, Montevideo, Uruguay; 9 Laboratorio de Interacciones Moleculares, Facultad de Ciencias, Universidad de la República, Montevideo, Uruguay

**Keywords:** peroxidase, Chagas disease, ascorbate, cytochrome c, heme, oxidants

## Abstract

The protozoan parasite *Trypanosoma cruzi* is the causative agent of American trypanosomiasis, otherwise known as Chagas disease. To survive in the host, the *T. cruzi* parasite needs antioxidant defense systems. One of these is a hybrid heme peroxidase, the *T. cruzi* ascorbate peroxidase-cytochrome *c* peroxidase enzyme (*Tc*APx-C*c*P). *Tc*APx-C*c*P has high sequence identity to members of the class I peroxidase family, notably ascorbate peroxidase (APX) and cytochrome *c* peroxidase (C*c*P), as well as a mitochondrial peroxidase from *Leishmania major* (*Lm*P). The aim of this work was to solve the structure and examine the reactivity of the *Tc*APx-C*c*P enzyme. Low temperature electron paramagnetic resonance spectra support the formation of an exchange-coupled [Fe(IV)=O Trp_233_^•+^] compound I radical species, analogous to that used in C*c*P and *Lm*P. We demonstrate that *Tc*APx-C*c*P is similar in overall structure to APX and C*c*P, but there are differences in the substrate-binding regions. Furthermore, the electron transfer pathway from cytochrome *c* to the heme in C*c*P and *Lm*P is preserved in the *Tc*APx-C*c*P structure. Integration of steady state kinetic experiments, molecular dynamic simulations, and bioinformatic analyses indicates that *Tc*APx-C*c*P preferentially oxidizes cytochrome *c* but is still competent for oxidization of ascorbate. The results reveal that *Tc*APx-C*c*P is a credible cytochrome *c* peroxidase, which can also bind and use ascorbate in host cells, where concentrations are in the millimolar range. Thus, kinetically and functionally *Tc*APx-C*c*P can be considered a hybrid peroxidase.

Chagas disease is named after the Brazilian physician Carlos Chagas, who first described it in 1909; its formal name is *American trypanosomiasis*. It most seriously affects isolated rural and indigenous communities with low economic development and little access to healthcare. The disease was once mainly confined to the American continent, principally Latin America, but the distribution of Chagas disease is expanding into Europe and North America and is becoming a public health issue at nonendemic sites (1, 2). The protozoan parasite *Trypanosoma cruzi* is the causative agent of the disease. It is estimated that around 10 million people across the whole of Latin America are infected with *T. cruzi*, causing *ca.* 20,000 deaths per annum. *T. cruzi* strains are heterogeneous, exhibiting a high degree of biochemical and genetic variability; this means that disease outcomes vary from asymptomatic during the course of infection to fatal in other cases (3).

In order to survive and proliferate in the host cells, the *T. cruzi* parasite needs antioxidant defense systems (4, 5) to cope against the cytotoxic effects of reactive oxygen and nitrogen species (6, 7, 8). One of these is a heme-containing peroxidase (9, 10). The enzyme is located in the endoplasmic reticulum and mitochondria in all parasite stages and is additionally located at the plasma membrane in the infective parasite stages (9). Originally designated as an ascorbate peroxidase (APX) (*Tc*APx (10)), this *T. cruzi* enzyme has high sequence identity to members of the class I peroxidase family. The class I peroxidases include APX and cytochrome *c* peroxidase (C*c*P), as well as a mitochondrial peroxidase from *Leishmania major* (*Lm*P). APX, C*c*P, and *Lm*P are all heme-dependent enzymes that scavenge, by reduction, hydrogen peroxide (H_2_O_2_) in cells and use ascorbate or cytochrome *c* as the reducing substrate (9, 11, 12, 13, 14). Like *Lm*P (15, 16), *Tc*APx was later found to have both ascorbate and cytochrome *c* activity (9) and was thus renamed as *Tc*APx-C*c*P. The parasite’s ability to utilize both ascorbate (which is endogenously synthesized from the host organism (17)) and cytochrome *c* as electron sources may be an evolutionary adaptation in these parasites, which have been present in some form since prehistoric times.

A lack of structural information for *Tc*APx-C*c*P has hindered the understanding of key enzymological aspects. Here, we present a crystal structure for *Tc*APx-C*c*P that, together with functional spectroscopic data, allows full rationalization of the substrate specificity and reactivity of this important enzyme target.

## Results

### Structure of the TcAPx-CcP enzyme

**Figure 1:**
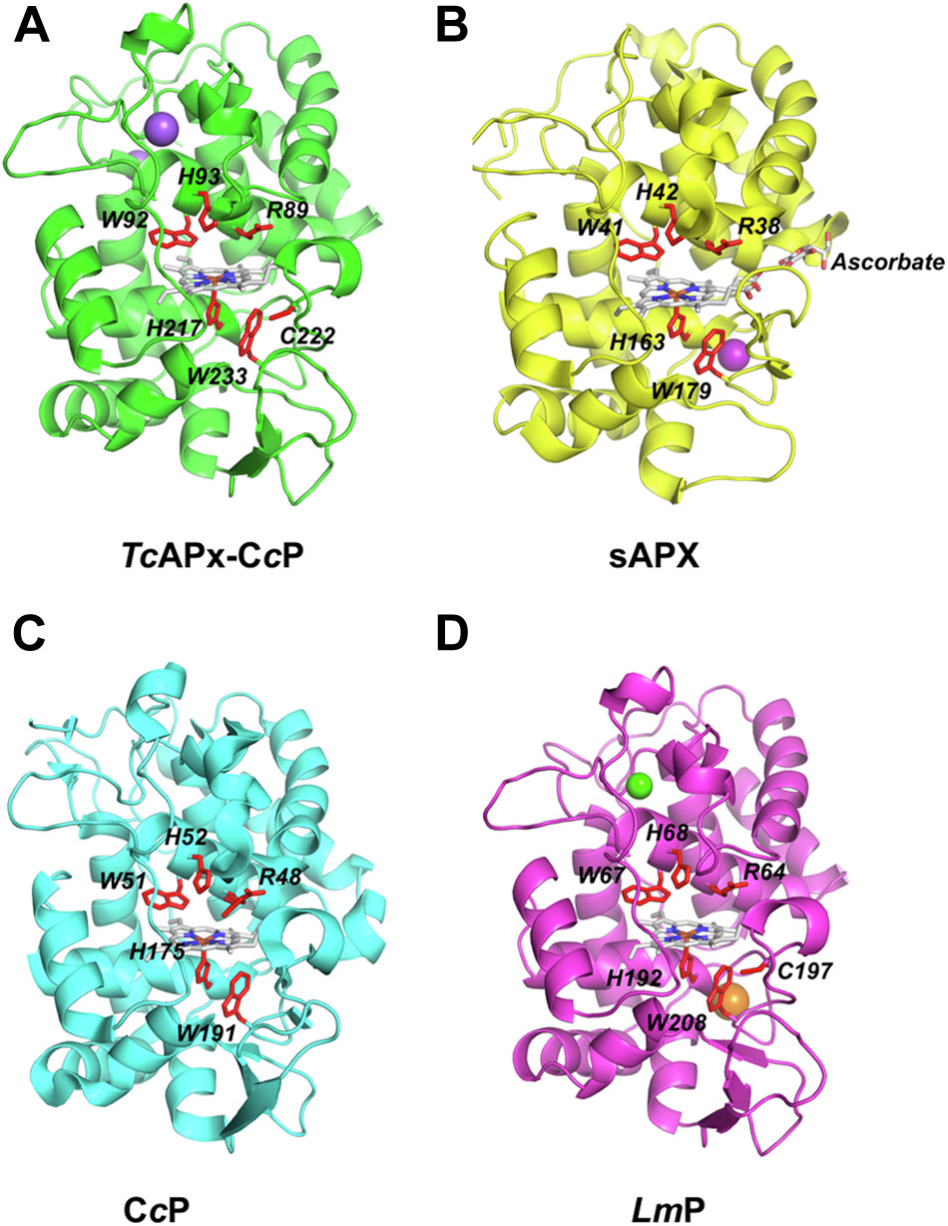
**Crystal structures of*Tc*APx-C*c*P and related heme peroxidases.***A*, *Tc*APx-C*c*P (PDB 7OPT). *B*, sAPX with ascorbate bound (1OAF). *C*, C*c*P (1ZBY). *D*, *Lm*P (3RIV). Active site residues are shown as *red sticks*. Sodium atoms are shown as *purple spheres*, calcium as *green spheres*, and potassium as *orange spheres*. APX, ascorbate peroxidase; C*c*P, cytochrome *c* peroxidase; PDB, Protein Data Bank.

**Figure 2:**
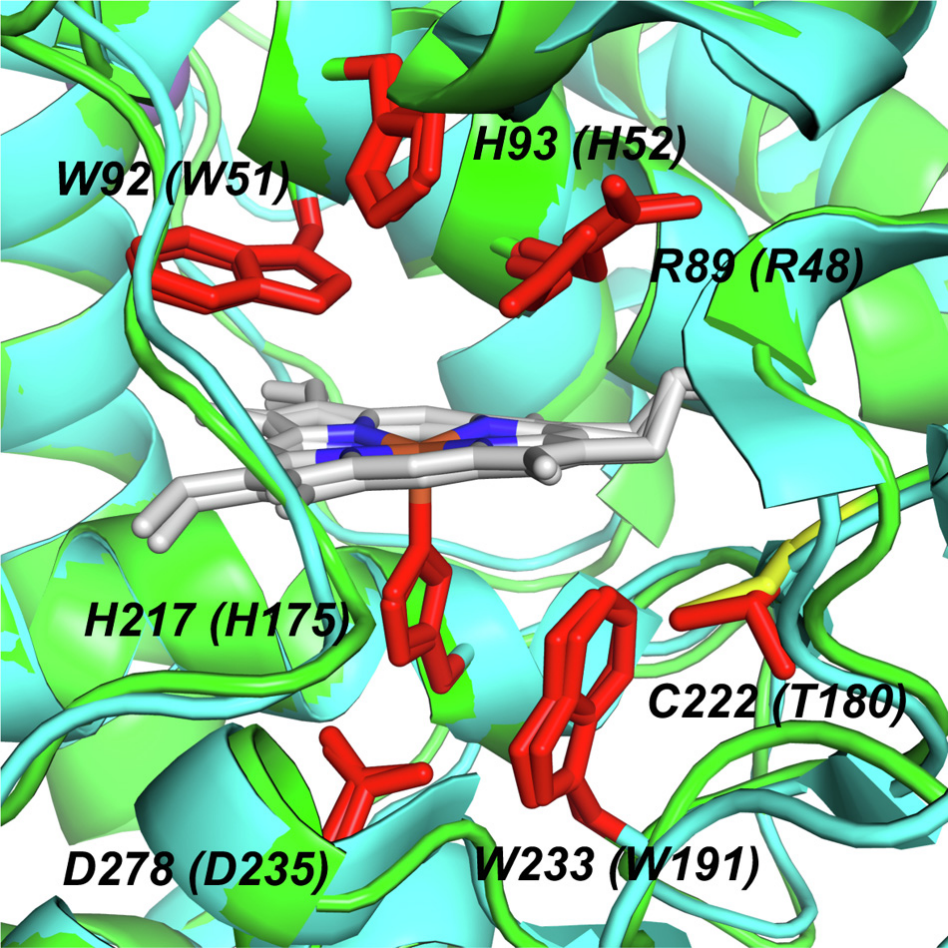
**The active site heme environment in *Tc*APx-C*c*P (in *green*) superimposed with C*c*P (*cyan*).** Active site residues are shown as *red sticks* and labeled for *Tc*APx-C*c*P with the equivalent residue in C*c*P in parentheses. *Tc*APx-CcP features the C222 residue (in *yellow*), whereas T180 is found in C*c*P (see Fig. S2). An equivalent of C222 is also found in *Lm*P (C197). C*c*P, cytochrome *c* peroxidase.

The structure of ferric *Tc*APx-C*c*P has been solved to 2.0 Å, Figure 1*A*; a comparison with the structures of related peroxidases is shown in Figure 1, *A–D*. Data and refinement statistics are shown in Table 1. The *Tc*APx-C*c*P enzyme is similar in its overall structure to the related *Lm*P enzyme with which *Tc*APx-C*c*P shares 57% sequence identity. The structure comprises of 10 α-helical bundles, which is consistent with the characteristics of other peroxidase structures. *Tc*APx-C*c*P also features a limited amount of ordered β-structure (located approximately between Gly250-Asn270), which has until now only been identified in C*c*P and to a slightly lesser extent *Lm*P (16). The active site of *Tc*APx-C*c*P comprises Trp92 in the distal pocket, along with distal histidine (His93) and arginine (Arg89) residues. On the proximal side there is a typical peroxidase His217-Asp278-Trp233 proximal triad, as observed in yeast C*c*P (Fig. 2) and in soybean APX (sAPX, His163-Asp208-Trp179, Fig. S2). There are two molecules in the asymmetric unit for both the wild type and mutant structures, each one exhibiting different electron density at the distal heme position, with one appearing to bind an oxygen molecule and another a water molecule approximately 2.3 Å above the iron (not shown). There are two metal cations, Figure 1*A*, most likely sodium from the crystallization conditions, located at ≈13 and ≈16 Å from the heme center near the solvent accessible outer edge of the structure.

**Table 1:** Data collection and refinement statistics for *Tc*APx-C*c*P (PDB code 7OPT) and W233F (7OQR)

Data collection	*Tc*APx-C*c*P	W233F
Resolution (Å)	62.02–2.02 (2.07–2.02)	29.28–1.76 (1.80–1.76)
Total measured reflections	987,780	1,516,631
Unique reflections	50,314 (3670)	76,005 (4170)
Completeness (%)	99.7 (99.1)	99.8 (97.3)
Redundancy	19.6 (20.5)	20 (19.6)
I/σ (I)	19.0 (2.4)	22.5 (4.1)
Unit cell dimensions (Å)	a = 71.6 b = 71.6 and c = 253.3	71.6, 71.6, 254.6
Space group	P3_1_21	P3_1_21
R_merge_	0.08 (1.4)	0.08 (0.74)
Refinement		
R_work_/R_free_	0.16/0.23	0.12/0.16
r.m.s.d. bond (Å)/angle (°)	0.01/1.8	0.01/1.9
*B*-factor analysis (Å^2^)		
Protein	49	27
Water	50	36
Ramachandran analysis		
Most favored (%)	94.94	97.58
Allowed (%)	5.06	2.05
Outliers Disallowed (%)	0.0	0.37

Values in parenthesis are for high resolution shells.

### Nature of the compound I intermediate

In C*c*P, reaction of the enzyme with H_2_O_2_ leads to formation of a protein radical located on Trp191 (18). This same Trp residue (Trp179) is not used in sAPX (19, 20, 21), despite the C*c*P and sAPX enzymes being essentially identical in the region of the proximal pocket. The different substrate specificities of the two enzymes—with ascorbate binding at the γ-heme edge in sAPX (22) and cytochrome *c* in C*c*P instead binding on an election transfer pathway that includes Trp191 (23)—is probably in part responsible for the difference in reactivity of the Trp residues between the two enzymes. The presence of a potassium cation on the proximal side of the heme in sAPX might also destabilize radical formation on Trp179 in APX (24, 25, 26), although we do not observe K^+^ cations in all of our sAPX structures (and there is no metal cation at the equivalent position in the *Tc*APx-C*c*P structure, as above).

**Figure 3:**
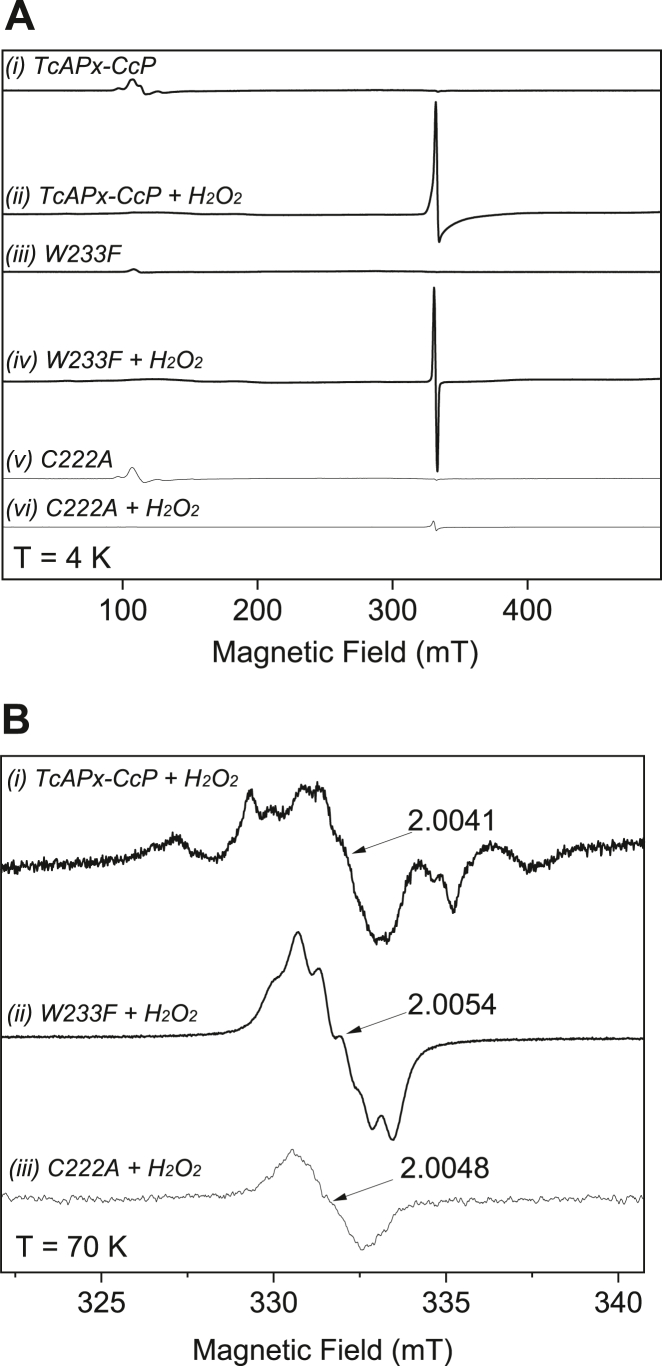
**Low-temperature EPR spectra of *Tc*APx-C*c*P.***A*, 9-GHz CW EPR spectra of (i) ferric *Tc*APx-C*c*P, (ii) compound I of *Tc*APx-C*c*P formed by reaction of the ferric sample with H_2_O_2_ for 10 s, (iii) ferric W233F, (iv) ferric W233F mixed with H_2_O_2_ for 10 s, (v) ferric C222A, and (vi) C222A mixed with H_2_O_2_ for 10 s. Spectra were recorded at 4.3 K, 4 G modulation amplitude, 1 mW microwave power, 100 kHz modulation frequency, two scans. *B*, (i) The same sample of compound I of *Tc*APx-C*c*P as in (*A*)(ii), (ii) The same sample as in (*A*)(iv), (iii) The same sample as in (*A*)(vi). Spectra in (*B*) were recorded at 70 K, 1 G modulation amplitude, 0.2 mW microwave power, 100 kHz modulation frequency, 100 scans. (ii) and (iii) have been multiplied by a factor of 6 to allow comparison. APX, ascorbate peroxidase; C*c*P, cytochrome *c* peroxidase.

Electron paramagnetic resonance (EPR) was used to assess whether *Tc*APx-C*c*P uses the corresponding Trp233 radical. EPR spectra taken at 4 K for *Tc*APx-C*c*P on reaction with H_2_O_2_ are consistent with the formation of a [Fe(IV)=O Trp^•+^] radical in compound I, with a concomitant reduction of the high spin iron heme, *g* = 6, signal (Fig. 3*A*i-ii). The radical was centered at *g* = 2 and showed a pronounced broad linewidth only present at the lowest temperatures with a narrower radical signal observed at 70 K (Fig. 3*B*(i)). This is consistent with the compound I intermediate containing an exchange coupled porphyrin-tryptophanyl radical, [Fe(IV)=O Por/Trp^•+^] at 4 K (18, 27). It is also consistent with the absorption spectra of *Tc*APx-C*c*P on reaction with H_2_O_2_ (9), which show peaks (539 and 560 nm) close to those observed for C*c*P (28, 29) and different from those for sAPX (which forms a porphyrin pi-cation radical instead (20, 21, 30)). Similar EPR spectra were reported (16) for the exchange coupled porphyrin-tryptophanyl radical in *Lm*P. This broadening was clearly absent in parallel experiments at 4 K on the W233F variant (Fig. 3*A*iii-iv). A structure of W233F, Fig. S4 and Table 1, shows no changes in the heme active site. In the case of the W233F variant, a narrow radical was observed at 4 K (Fig. 3*A*(iii-iv), which persisted at 70 K (Fig. 3*B*(ii)) with a *g* value of 2.0048. This is similar to the W191F variant of C*c*P that forms a tyrosyl radical (31).

Formation of a cysteinyl radical has previously been proposed for *Tc*APx-C*c*P (9). The EPR spectra of *Tc*APx-C*c*P on reaction with H_2_O_2_ at 70 K (Fig. 3*B*(i)) shows resonances with *g* values centered at 2.0041, again consistent with the formation of amino acid radical/s (32). Cysteinyl radicals are known to have an axially symmetric *g*-tensor with *g*_x_ values of 2.16 to 2.5 (32, 33, 34); we do not clearly observe this feature, although the *g*_x_ line is known to be weak (33, 34). The spectral features at *g* = 2.0041 span a width of ∼12.5 mT, which is consistent with that observed previously for cysteinyl radicals (33, 34). This is not typical of isolated tyrosyl and tryptophanyl radicals, which have narrower spectral widths (31, 35). When the EPR experiments were repeated for the C222A variant (Fig. 3*A*(v-vi)), a signal with *g* value of 2.0048 was observed at 4 K and persisted at 70 K (Fig. 3*B*(iii)), consistent with either tyrosine (36) or tryptophan (31, 35) radicals. Formation of amino acid radicals observed at higher temperatures is often observed in off-pathway processes in heme proteins (37, 38). The radical/s generated from C222A mutant were notably narrower (Fig. 3*B*(iii)) than that found for *Tc*APx-C*c*P. We tentatively interpret the spectrum shown in Figure 3*B*(i) as evidence of cysteinyl radical formation in *Tc*APx-C*c*P, which is consistent with the previous assignment using spin trapping assays (9). Further multifrequency EPR work would be needed to unambiguously identify these radical species. Corresponding changes in absorbance spectra for the C222A mutant are shown in Fig. S5.

### Substrate specificity

**Figure 4:**
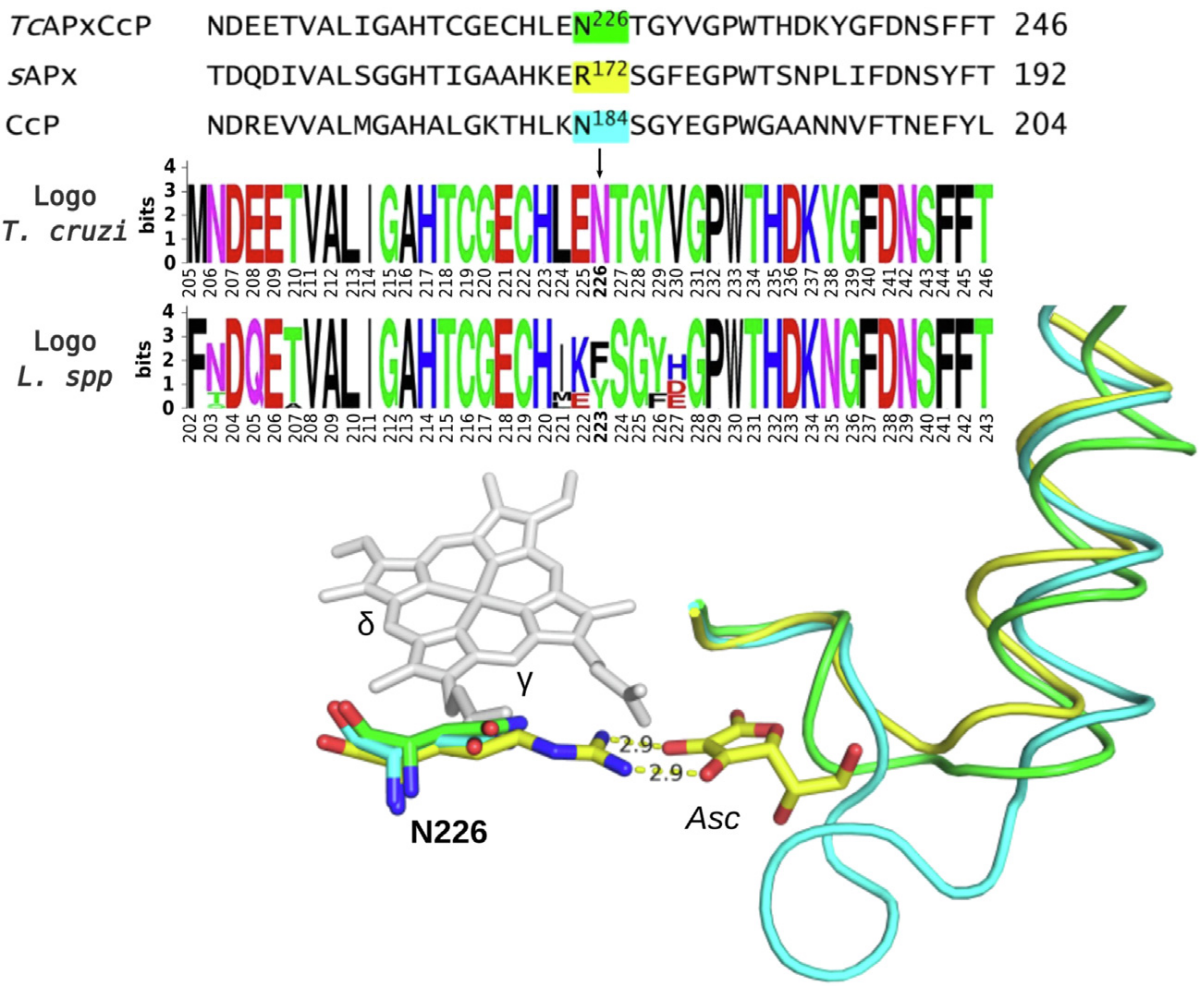
**Comparison of the structures of the γ-heme edge of *Tc*APx-C*c*P (*green*), sAPX (*yellow*), and yeast C*c*P (*cyan*).** Ascorbate bound to sAPX is shown as *yellow sticks*. The important R172 ascorbate-binding residue in APX is shown superimposed with the equivalent residues (N226, N184) from *Tc*APx-C*c*P and C*c*P, respectively. The relevant sequence in this region is also shown, along with sequence logos obtained from *Trypanosoma cruzi* and *Leishmania spp.* alignments (see also Figs. S2 and S3). The extended loop which might interfere with ascorbate binding (22) is clearly visible in C*c*P but missing in *Tc*APx-C*c*P. Hydrogen bonds and their distances between R172 of sAPX and ascorbate are indicated with *yellow dashes*. APX, ascorbate peroxidase; C*c*P, cytochrome *c* peroxidase.

In sAPX, the ascorbate substrate is hydrogen bonded to Arg172 at the γ-heme edge (22). No equivalent Arg residue is present in *Tc*APx-C*c*P, and this residue is instead replaced with Asn226, which is fully conserved in every *T. cruzi* genome evaluated (Figs. 4 and S2). Interestingly, this position is occupied by tyrosine or phenylalanine in *Leishmania* related species (Fig. 4). The ordered water molecules found in this region in sAPX are not present in *Tc*APx-C*c*P (not shown). Arg172 in sAPX is essential for ascorbate activity (39). Lys30, which also interacts with the bound ascorbate in the sAPX-ascorbate complex, is also missing in *Tc*APx-C*c*P and is replaced with Glu79 and Asp80 (see also later). In C*c*P, an ascorbate-binding site can be engineered into the enzyme (40) by inclusion of an Arg residue at the appropriate location (N184R mutation in C*c*P, Figs. 4 and S2). Ascorbate binding and catalytic activity is weak (*k*_cat_ = 1.5 s^−1^; *K*_M_ = 1.7 mΜ) but detectable in this N184R variant of C*c*P (40), when compared to wild type sAPX (*k*_cat_ = 272 s^−1^; *K*_M_ = 389 μM; *k*_cat_/*K*_M_ = 0.69 μM^−1^s^−1^ (20)). The equivalent N226R mutation in *Tc*APx-C*c*P (equivalent to N184R in C*c*P) is viable in terms of protein expression and protein stability but showed very weak heme binding. It was therefore not possible to measure ascorbate activity for this variant. This indicates that building an ascorbate binding at this site is not viable, even in the presence of the required Arg residue. Attempts to crystallize *Tc*APx-C*c*P in complex with ascorbate were unsuccessful.

**Figure 5:**
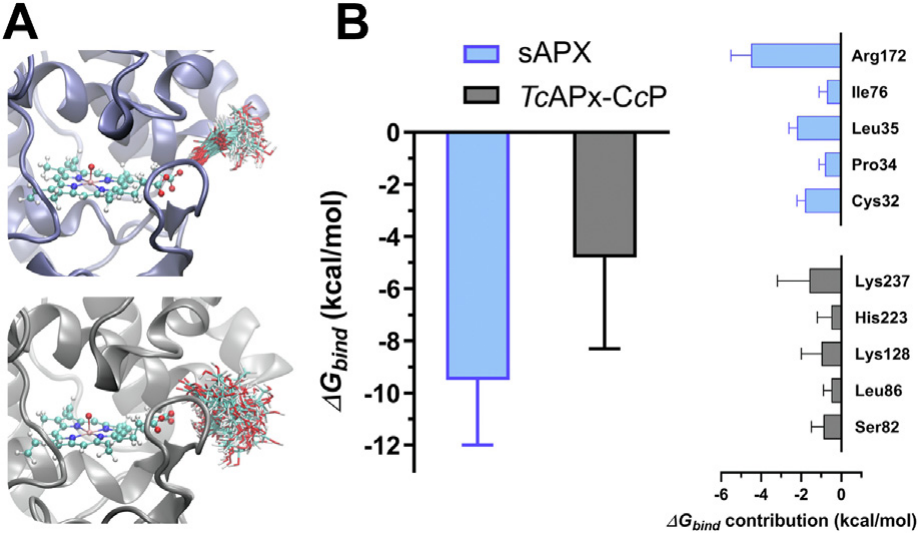
**Interaction of ascorbate at the active site of APX evaluated by MD simulation.***A*, superimposition of the conformations adopted by ascorbate at the active site of *s*APX (*blue cartoon*, up) and *Tc*APx-C*c*P (*black cartoon*, down). *B*, ascorbate binding free energy (*ΔG*_*bind*_, kcal/mol) estimation using the MM-GBSA formalism, along with a residue basis decomposition of the free energy. Only residues contributing more than 0.5 kcal/mol to the calculated binding free energy are shown. APX, ascorbate peroxidase; C*c*P, cytochrome *c* peroxidase; MD, molecular dymamics.

To better understand the interaction of ascorbate with *Tc*APx-C*c*P, we performed molecular dynamics (MD) simulations of both the sAPX–ascorbate and *Tc*APx-C*c*P–ascorbate complexes. The initial ascorbate pose in the *Tc*APx-C*c*P active site was assumed from the sAPX–ascorbate crystal structure (22) and then minimized prior to MD simulations (see Experimental procedures). An initial inspection of ascorbate dynamical behavior in both active sites showed significant differences between these complexes; while the interactions displayed by ascorbate with sAPX are specific and long-lived, leading to one preferential ascorbate conformation, in the case of the *Tc*APx-C*c*P–ascorbate complex, those interactions are not as strong and a myriad of ascorbate orientations were observed (Fig. 5*A*). Using the MM-GBSA formalism, we estimated ascorbate binding free energies (*ΔG*_*bind*_) for both cases, which shows a significantly lower ascorbate *ΔG*_*bind*_ for the *s*APX case (Fig. 5*B*), that is, stronger substrate interaction with the enzyme. Furthermore, a residue basis decomposition of the estimated *ΔG*_*bind*_ values indicates a major role for Arg172 in stabilizing the *s*APX–ascorbate complex, explaining a very significant portion of the observed difference in *ΔG*_*bind*_ values (Fig. 5*B*). Lys237, likely to be the most important residue in stabilizing the *Tc*APx-C*c*P–ascorbate interaction (Fig. 5*B*), is fully conserved both in *T. cruzi* and *Leishmania spp.* (see Fig. S3).

### Complex formation with cytochrome c

**Figure 6:**
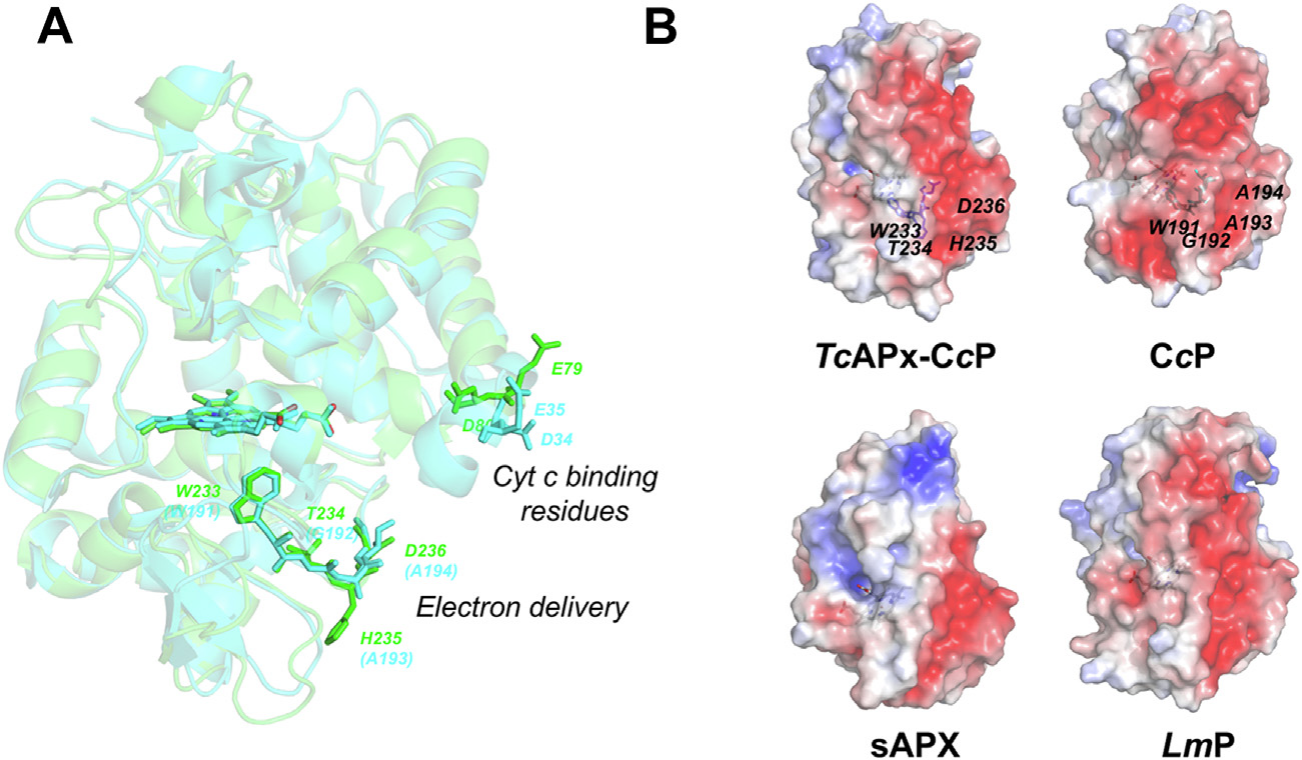
**Comparison of electron transfer pathways and surface electrostatics.** A, alignment of the structures of *Tc*APx-C*c*P (*faded green*) and C*c*P (*faded cyan*). The residues involved in the delivery of electrons from cytochrome *c* in C*c*P (Trp191, Gly192, Ala193, and Ala194, in *green*) overlay well with an equivalent electron pathway in *Tc*APx-C*c*P (Trp233, Thr234, His235, and Asp236, in *green*). The heme group is shown for both proteins. The residues involved in binding of cytochrome *c* in C*c*P (Asp34, Glu35 (23)) overlay well in the *Tc*APx-C*c*P structure (Glu79 and Asp80, respectively). *B*, electrostatic surface representation of *Tc*APx-C*c*P (±5 kT), C*c*P, sAPX, and *Lm*P, obtained using the APBS software (63). The predicted cyt *c* binding surface is represented in *red* where the overall charge is strongly electronegative. This electronegative area is substantially less prominent in sAPX; sAPX does not bind cyt *c.* The residues in *Tc*APx-C*c*P which are expected to be responsible for the electron transfer path are shown as *dark blue sticks* (W233, T234, H235, and D236). The equivalent residues (W191, G192, A193, and A194) in C*c*P are shown as *cyan sticks* in (*B*). See Fig. S2 for a sequence alignment highlighting these residues. APX, ascorbate peroxidase; C*c*P, cytochrome *c* peroxidase.

Cytochrome *c* has a cluster of positively charged lysine residues on the surface, which facilitates its binding to the overall negatively charged surfaces of C*c*P (41, 42), Figure 6*A*. We were not able to obtain a crystal structure of *Tc*APx-C*c*P in complex with cytochrome *c*, but we carried out an analysis of the electrostatic surface charges of *Tc*APx-C*c*P, C*c*P, APX, and *Lm*P, Figure 6. The surface electrostatics of *Tc*APx-C*c*P more closely resemble those of C*c*P than they do sAPX, Figure 6*B*, with *Tc*APx-C*c*P showing a broad negatively charged region on the surface where cytochrome *c* would be expected to bind. The similarity is even more striking when comparing *Tc*APx-C*c*P to *Lm*P, Figure 6*B*; *Lm*P has been categorized as a C*c*P-like enzyme (43).

## Discussion

The denomination of the *Tc*APx-C*c*P enzyme is derived from its relationship to two other peroxidase enzymes with which *Tc*APx-C*c*P shares sequence similarities: APX and C*c*P. APX and C*c*P have different substrate binding properties—APX binds to and sources its electrons from a small molecule, ascorbate, whereas C*c*P transiently interacts with another protein reductase, cytochrome *c*. Both APX and C*c*P function through a mechanism that involves formation of a high-valent (oxidized) heme species, known as compound I, as shown below (P = peroxidase):

P + H_2_O_2_ → Compound I +H_2_O

Compound I + HS → Compound II + S^•^

Compound II + HS → P + S^•^ + H_2_O

A crucial distinction is that C*c*P uses a highly atypical protein-based radical (located on Trp191 (18)) in its compound I, rather than the usual porphyrin-π-cation radical used in APX (19, 20, 21) and all other peroxidases (44). While APX and C*c*P are structurally very similar, with a well-conserved fold, there are notable differences in the substrate-binding regions that help to account for the different substrate-binding behaviors (22, 39). In contrast, the lack of structural information for *Tc*APx-C*c*P has made interpretation of its reactivity more difficult.

The information presented in this article now substantially clarify the properties and catalytic behavior of the *Tc*APx-C*c*P enzyme. The active site of *Tc*APx-C*c*P closely resembles that of APX, C*c*P, and *Lm*P. The low temperature EPR spectra support the formation of a reactive compound I intermediate with an exchange-coupled [Fe(IV)=O Trp_233_^•+^] radical. If the presence of a cation on the proximal side of the heme has a role in destabilizing radical formation on Trp (24, 25, 26), then the absence of such a cation in the *Tc*APx-C*c*P structure is logical. All the EPR spectra at 70 K showed evidence of the generation of amino acid radicals on reaction of *Tc*APx-C*c*P with H_2_O_2_. The spectral features in the case of the wild type *Tc*APx-C*c*P enzyme were consistent with the generation of a cysteinyl radical, as suggested previously (9).

Based on the sequence and the new structural and MD simulation information, *Tc*APx-C*c*P would not be expected to be highly competent for ascorbate binding at the γ-heme edge because, like C*c*P (40), it lacks the key Arg residue (Arg172 in APX) that is required for ascorbate binding (39), Figures 4 and 5. It also lacks Lys30, which is replaced by a pair of negatively charged residues (Glu79, Asp80) as found in C*c*P. However, *Tc*APx-C*c*P also lacks the extended loop that is present in C*c*P in the γ-heme–binding region, and this loop probably prevents ascorbate binding in C*c*P (22). This might work in favor of ascorbate binding at this location, but we were not able to detect ascorbate at this site in the structure. While it is possible to re-engineer ascorbate activity into C*c*P by introduction of an Arg in the correct location (N184R mutation (40)), this was not successful for in *Tc*APx-C*c*P (N226R). On this basis, it is difficult to conclude that *Tc*APx-C*c*P is a bona fide APX. The higher catalytic efficiency of *Tc*APx-C*c*P for cytochrome *c* (*k*_cat_/*K*_M_ = 2.1 × 10^5^ M^−1^s^−1^) than for ascorbate (*k*_cat_/*K*_M_ = 3.5 × 10^4^ M^−1^s^−1^, Table 2) (9) supports this conclusion. Ascorbate activity for sAPX is around 10-fold higher (*k*_cat_/*K*_M_ = 6.9 × 10^5^ M^−1^s^−1^ (20)) than that for *Tc*APx-C*c*P; C*c*P is itself a very poor APX (*k*_cat_/*K*_M_ = 1.1 × 10^3^ M^−1^s^−1^ for ascorbate oxidation (40)), Table 2.

**Table 2:** Summary of steady state data for ascorbate oxidation for various peroxidases

Enzyme	Ascorbate			Cytochrome *c*			Ref
	*k*_cat_ (s^−1^)	*K*_M_ (μM)	*k*_cat_/*K*_M_ (M^−1^s^−1^)	*k*_cat_ (s^−1^)	*K*_M_ (μM)	*k*_cat_/*K*_M_ (M^−1^s^−1^)	
*Tc*APx-C*c*P^a^	0.067	190	3.5 × 10^4^	6.1	29	2.1 × 10^5^	(9)
APX^b^	272	389	6.9 × 10^5^	-	-	-	(20)
C*c*P	0.83	710	1.1 × 10^3^	150	93	1.6 × 10^6^	(40)
*Lm*P	-	-	-	1700	8	2 × 10^8^	(16)

a The W233F variant of *Tc*APX-C*c*P has no measurable activity for cytochrome *c* and exhibits a 10-fold decrease in ascorbate activity compared to the wild type enzyme (9).

b APXs have no measurable activity for cytochrome *c* (12, 64).

The known electron transfer pathway from cytochrome *c* in the C*c*P/c complex (23) is replicated in *Tc*APx-C*c*P, Figure 6*A*. Whilst we were unable to obtain a structure for the *Tc*APx-C*c*P-cytochrome *c*, a comparison of surface electrostatics, Figure 6*B*, is compelling in its similarity to both C*c*P and *Lm*P, the latter of which it shares a 57% sequence identity (Fig. S2). *Tc*APx-C*c*P contains the residues equivalent to Asp34 and Glu35 in C*c*P (Glu79 and Asp80, Fig, S2), which align well in the structure, Figure 6. These residues, along with Asp37 and Glu290 in C*c*P (which are absent in *Tc*APx-C*c*P, Fig. S2), are important for cytochrome *c* binding (23) and indeed are entirely absent in sAPX.

The structural information and bioinformatic analysis provided herein are consistent with an assignment of *Tc*APx-C*c*P as a credible cytochrome *c* peroxidase but a poorer APX and in agreement with other conclusions for *Lm*P (15, 16). The steady state kinetic information, when compared across all three enzymes (*Tc*APx-C*c*P, APX, and C*c*P) and *Lm*P, bears this out. Having said that, while ascorbate activity is evidently weaker in *Tc*APx-C*c*P, it would not be surprising for a C*c*P-like enzyme to exhibit some level of ascorbate-dependent activity given the structural similarities of the C*c*P, APX, and *Tc*APx-C*c*P enzymes. Peroxidases are not fussy enzymes in terms of their substrate specificity—they can usually oxidize several different substrates and can bind the same substrate at several different locations (20, 45). Even APXs, while presumably designed to favor ascorbate binding, might bind ascorbate at other (unknown) locations (20) (and can oxidize numerous other substrates too). This could well be the case here for *Tc*APx-C*c*P, with ascorbate binding at one of several nonspecific locations (at, or remote from, the γ-heme edge) and thus retaining some ascorbate activity (9). This would be beneficial to the parasite at the plasma membrane in infective extracellular trypomastigotes and intracellular amastigotes (9), as millimolar concentrations of ascorbate are known to be present in the mammalian extracellular milieu or host cell cytoplasm (46, 47). Thus, *Tc*APX can be considered kinetically, evolutionarily, and functionally as a hybrid peroxidase. While is clear that it is less finely tuned for oxidation of ascorbate in comparison to sAPX, it still exhibits reasonable *K*_M_ and *k*_cat_/*K*_M_ (see comparative values in Table 2). Thus, *Tc*APX likely binds and utilizes ascorbate opportunistically and in an unoptimized manner in host cells as an evolutionary adaptation.

## Experimental procedures

### Solutions

All assays, unless specified, were performed in potassium phosphate buffer 50 mM pH 7.4. The concentrations of *Tc*APx-C*c*P and H_2_O_2_ were spectrophotometrically determined at 409 nm (101 mM^−1^ cm^−1^) and 240 nm (39.4 M^−1^ cm^−1^ (48)), respectively.

### Expression and purification

The plasmid for expression of *Tc*APx-C*c*P in *Escherichia coli* was provided by Shane Wilkinson, Queen Mary University, UK (10) in the pTrcHis expression vector. Purification was carried out as previously described (9, 10) except that δ-aminolevulinic acid (0.5 mM) was added at the point of inducing expression with IPTG (0.8 mM) in order to improve the incorporation of heme into the protein. Terrific broth growth medium was used for all expressions. Purity of protein preparations was assessed by a 12.5 % SDS-PAGE gel and the relative height of the 409 nm Soret peak (ε_409 nm_ = 101 mM^−1^ cm^−1^) to the overall protein content peak according to the 280 nm signal (ε_280 nm_ = 56.9 mM^−1^ cm^−1^). Typically, after a nickel affinity column, a range of fractions of varying purity (Fig. S1) were obtained. For kinetics experiments, only the purest samples were used (as judged by SDS-gel and the A_Soret_/A_280_ ratio). For crystallography experiments, an additional purification step was carried out using a Superdex 75 gel filtration column on an FPLC instrument. For all experiments, the protein concentration was determined spectrophotometrically using the 409 nm Soret peak. Percentage heme incorporation in purified samples was calculated from the *A*_409_/*A*_280_ ratio, with the holoenzyme representing typically *ca* 60%. The concentration as determined from the 409 nm signal representing holoprotein was used exclusively in all subsequent experiments.

Site-directed variants (C222A and N226R) were produced according to the KLD enzyme mix protocol (New England Biolabs). Mutations were confirmed by DNA sequencing by Eurofins Genomics using pTrcHis forward and reverse standard primers. The purification protocol for the variants was the same as for the WT protein except for N226R, which required the addition of free heme before gel filtration to increase the proportion of holoprotein in the sample.

### Crystallography

*Tc*APx-C*c*P was purified as described previously and was concentrated to 9 mg/ml. Initial crystals of both WT and the W233F variant were obtained using an Art Robbins Phoenix/Gryphon crystallography robot and Molecular Dimensions screens. The best crystals were grown in 2 μl of reservoir solution (2.0 M ammonium sulfate, 0.1 M sodium acetate pH 4.6) and an equal amount of protein solution using the hanging drop method. Irregularly sized, hexagonal prism–shaped crystals grew to their maximum size within a week at 18 °C. Crystals of holoprotein were soaked in a cryoprotectant solution consisting of the reservoir solution plus 25% glycerol and then flash frozen in liquid nitrogen prior to data collection.

Data collection was carried out at the Diamond Light Source I03. Data were indexed using iMOSFLM and then scaled and merged using AIMLESS as part of the CCP4 suite. The protein crystallized with two molecules per asymmetric unit and belongs to the P 3_1_ 2 2 trigonal space group with unit cell dimensions of a = 71.6 Å, b = 71.6 Å, and c = 253.3 Å. The crystal was found to be twinned and was refined as such. Data collection and refinements statistics are shown in Table 1. The structure was determined by molecular replacement using the *Lm*P structure (Protein Data Bank 3RIV) as the search model in Phaser (49) and refined with REFMAC (50) and COOT (51). The average B factor for the WT *Tc*APx-C*c*P is significantly higher than for the W233F variant (50 vs 26), which is indicative of relatively higher mobility in WT. Attempts to crystallize *Tc*APx-C*c*P in complex with either ascorbate or cytochrome *c* were unsuccessful.

### EPR

Samples (75–100 μM, 50 mM sodium phosphate buffer, pH 7.4) were prepared by mixing an equal volume of hydrogen peroxide with *Tc*APx-C*c*P and reacting for 10 s before being flash frozen into 4 mm quartz EPR tubes. Continuous-wave EPR spectra at X band (9 GHz) were recorded using a Bruker EMX spectrometer. The spectrometer was equipped with a super high sensitivity probe head and a liquid helium cryostat (Oxford Instruments). Typical X-band spectra were recorded under nonsaturating conditions at 4 and 70 K to ascertain unique species. EPR parameters are stated on the Figure legends. *g*-values were calibrated against a 2,2-diphenyl-1-picrylhydrazyl standard.

### Bioinformatic data retrieval and analysis

Amino acid sequences of *T. cruzi* CL Brener Esmeraldo–like *Tc*CLB.503745.30 (328 aa) were obtained from TriTrypDB version 56 (52) and used as query for a BLASTp search (53) in *T. cruzi* and *Leishmania* species. Seventy-two APX sequences were obtained with e-values > 8,00E-06: 58 sequences from 18 *Leishmania* species (*Leishmania enriettii*, *Leishmania ghana*, *Leishmania namibia*, *Leishmania aethiopica*, *Leishmania amazonensis*, *Leishmania arabica*, *Leishmania donovani*, *Leishmania gerbilli*, *Leishmania infantum*, *L. major*, *Leishmania martiniquensis*, *Leishmania tarantolae*, *Leishmania mexicana*, *Leishmania braziliensis*, *Leishmania turanica*, *Leishmania tropica*, *Leishmania orientalis*, and *Leishmania panamensis*) and 16 sequences from nine *T. cruzi* strains (*T. cruzi Brazil A4*, *T. cruzi CL Brener* [*Esmeraldo Like and Non-Esmeraldo Like haplotypes*], *T. cruzi Dm28c, T. cruzi marinkellei, T. cruzi CL, T. cruzi G, T. cruzi TCC*, *T. cruzi Sylvio*, and *T. cruzi Y*). In addition, BLASTp was performed against *Glycine max* and *Saccharomyces cerevisiae* to get homologous sequences from those species (IDs Q43758 from UniProt and AJS52974.1 from the NCBI database, respectively). First 73 amino acids from the *S. cerevisiae* C*c*P sequence, corresponding to mitochondrial signal peptide, were removed to perform the subsequent analysis.

Multiple sequence alignment was performed using MUSCLE (54). Alignments were manually curated removing partial sequences. Finally, sequence logos (55) were made (that is, graphical representations of the sequence conservation of amino acids) for *T. cruzi* and *Leishmania* species alignments, using the web-based application WebLogo (56). Briefly, the sequence conservation at a particular position R_seq_ is defined as the difference between the maximum possible entropy (S_max_) and the entropy of the observed symbol distribution (S_obs_):

$$R_{seq}=S_{\max}-S_{obs}=log_{2}N-\left(\overset{N}{\underset{n=1}{\sum }}p_{n}log_{2}p_{n}\right),$$

where $p_{n}$ is the observed frequency of symbol n at a particular position and N is the number of distinct symbols for the given sequence type (20 for protein). Consequently, the maximum sequence conservation per site is log_2_ (20)≈4 bits (55).

### Classical MD and binding free energy estimations

MD simulations of both sAPX and *Tc*APx-CcP dimeric structures were performed, using as starting models the sAPX (1OAF (22)) and *Tc*APx-C*c*P (7OPT, this work) structures, respectively. The concomitant ascorbate complexes (sAPX–ascorbate and *Tc*APx-C*c*P–ascorbate) were simulated. The initial ascorbate pose in the *Tc*APx-C*c*P active site was generated by a structural alignment of the sAPX–ascorbate structure and that of *Tc*APx-C*c*P, followed by an energy minimization of the ascorbate moiety. These four systems assumed the heme to be in the oxyferryl state and were subjected to the same MD protocol. Briefly, systems were solvated using a default method, with an octahedral box of 12 Å in radius with TIP3P water molecules (57). Protein residue parameters correspond to the parm14SB Amber force field (58), oxyferryl-heme parameters correspond to the previously developed ones (9, 59), and ascorbate parameters were developed by a standard procedure: partial charges were computed using the restricted electrostatic potential recipe and density functional theory based electronic structure calculations with the Perdew-Burke-Ernzerhof functional and *dzvp* basis set. Equilibrium distances and angles, as well as force constants, were computed using the same methods and the basis set used for computed charges. All simulations were performed using periodic boundary conditions with a 10 Å cutoff and particle mesh Ewald summation method for treating the electrostatic interactions. The hydrogen bond lengths were kept at their equilibrium distance by using the SHAKE algorithm, while temperature and pressure were kept constant with a Langevin thermostat and barostat, respectively, as implemented in the AMBER program (60). In every case, the system was optimized in 1000 steps (10 with steep gradient and the rest with conjugate gradient). Then, it was slowly heated from 0 K to 300 K for 20 ps at constant pressure, with Berendsen thermostat, and pressure was equilibrated at 1 bar for 5 ps. After these two steps, a 10 ns MD long simulation at constant temperature (300 K) and constant volume was performed. Afterward, 500 ns trajectories in which a “wall-like” restraint was applied, biasing carboxylic groups from heme and ascorbate species distance to be less than 5 Å was performed. Binding free energy calculations were performed at the molecular mechanics/generalized Born and surface area level (61), selecting 500 representative equally spaced structures from each trajectory for analysis. The residue basis free energy decomposition among the closest 25 protein residues from ascorbate was calculated. All dynamics visualizations and molecular drawings were performed with VMD 1.9.1 (62).

## Data availability

Atomic coordinates have been deposited in the Protein Data Bank (PDB ID codes 7OPT [*Tc*APx-C*c*P] and 7OQR [W233F *Tc*APx-C*c*P]).

## Supporting information

This article contains supporting information.

## Conflict of interest

The authors declare that they have no conflicts of interest with the contents of this article.

## References

[1] Bonney K.M. (2014). Chagas disease in the 21st century: a public health success or an emerging threat?. Parasite.

[2] Bern C., Kjos S., Yabsley M.J., Montgomery S.P. (2011). Trypanosoma cruzi and Chagas' disease in the United States. Clin. Microbiol. Rev..

[3] Luquetti A.O., Miles M.A., Rassi A., de Rezende J.M., de Souza A.A., Povoa M.M. (1986). Trypanosoma cruzi: zymodemes associated with acute and chronic Chagas' disease in central Brazil. Trans. R. Soc. Trop. Med. Hyg..

[4] Piacenza L., Peluffo G., Alvarez M.N., Martinez A., Radi R. (2013). Trypanosoma cruzi antioxidant enzymes as virulence factors in Chagas disease. Antioxid. Redox Signal..

[5] Piacenza L., Trujillo M., Radi R. (2019). Reactive species and pathogen antioxidant networks during phagocytosis. J. Exp. Med..

[6] Martinez A., Prolo C., Estrada D., Rios N., Alvarez M.N., Pineyro M.D. (2019). Cytosolic Fe-superoxide dismutase safeguards Trypanosoma cruzi from macrophage-derived superoxide radical. Proc. Natl. Acad. Sci. U. S. A..

[7] Estrada D., Specker G., Martinez A., Dias P.P., Hissa B., Andrade L.O. (2018). Cardiomyocyte diffusible redox mediators control trypanosoma cruzi infection: role of parasite mitochondrial iron superoxide dismutase. Biochem. J..

[8] Alvarez M.N., Peluffo G., Piacenza L., Radi R. (2011). Intraphagosomal peroxynitrite as a macrophage-derived cytotoxin against internalized trypanosoma cruzi: consequences for oxidative killing and role of microbial peroxiredoxins in infectivity. J. Biol. Chem..

[9] Hugo M., Martinez A., Trujillo M., Estrada D., Mastrogiovanni M., Linares E. (2017). Kinetics, subcellular localization, and contribution to parasite virulence of a Trypanosoma cruzi hybrid type A heme peroxidase (TcAPx-CcP). Proc. Natl. Acad. Sci. U. S. A..

[10] Wilkinson S.R., Obado S.O., Mauricio I.L., Kelly J.M. (2002). Trypanosoma cruzi expresses a plant-like ascorbate-dependent hemoperoxidase localized to the endoplasmic reticulum. Proc. Natl. Acad. Sci. U. S. A..

[11] Sharp K.H., Moody P.C.E., Raven E.L. (2003). Defining substrate specificity in haem peroxidases. Dalton Trans..

[12] Raven E.L. (2003). Understanding functional diversity and substrate specificity in haem peroxidases: what can we learn from ascorbate peroxidase?. Nat. Prod. Rep..

[13] Raven E.L., Holzenberg A., Scrutton N.S. (2000). Subcellular Biochemistry: Enzyme Catalysed Electron and Radical Transfer.

[14] Adak S., Datta A.K. (2005). Leishmania major encodes an unusual peroxidase that is a close homologue of plant ascorbate peroxidase: a novel role of the transmembrane domain. Biochem. J..

[15] Jasion V.S., Doukov T., Pineda S.H., Li H., Poulos T.L. (2012). Crystal structure of the Leishmania major peroxidase-cytochrome c complex. Proc. Natl. Acad. Sci. U. S. A..

[16] Jasion V.S., Polanco J.A., Meharenna Y.T., Li H., Poulos T.L. (2011). Crystal structure of Leishmania major peroxidase and characterization of the compound I tryptophan radical. J. Biol. Chem..

[17] Wilkinson S.R., Prathalingam S.R., Taylor M.C., Horn D., Kelly J.M. (2005). Vitamin C biosynthesis in trypanosomes: a role for the glycosome. Proc. Natl. Acad. Sci. U. S. A..

[18] Sivaraja M., Goodin D.B., Smith M., Hoffman B.M. (1989). Identification by endor of Trp191 as the free-radical site in cytochrome c peroxidase compound ES. Science.

[19] Lad L., Mewies M., Basran J., Scrutton N.S., Raven E.L. (2002). Role of histidine 42 in ascorbate peroxidase. Kinetic analysis of the H42A and H42E variants. Eur. J. Biochem..

[20] Lad L., Mewies M., Raven E.L. (2002). Substrate binding and catalytic mechanism in ascorbate peroxidase: evidence for two ascorbate binding sites. Biochemistry.

[21] Patterson W.R., Poulos T.L., Goodin D.B. (1995). Identification of a porphyrin pi-cation-radical in ascorbate peroxidase compound-I. Biochemistry.

[22] Sharp K.H., Mewies M., Moody P.C., Raven E.L. (2003). Crystal structure of the ascorbate peroxidase-ascorbate complex. Nat. Struct. Biol..

[23] Pelletier H., Kraut J. (1992). Crystal-structure of a complex between electron-transfer partners, cytochrome c peroxidase and cytochrome c. Science.

[24] Bonagura C.A., Bhaskar B., Sundaramoorthy M., Poulos T.L. (1999). Conversion of an engineered potassium-binding site into a calcium-selective site in cytochrome c peroxidase. J. Biol. Chem..

[25] Bonagura C.A., Sundaramoorthy M., Bhaskar B., Poulos T.L. (1999). The effects of an engineered cation site on the structure, activity, and EPR properties of cytochrome c peroxidase. Biochemistry.

[26] Bonagura C.A., Sundaramoorthy M., Pappa H.S., Patterson W.R., Poulos T.L. (1996). An engineered cation site in cytochrome c peroxidase alters the reactivity of the redox active tryptophan. Biochemistry.

[27] Houseman A.L.P., Doan P.E., Goodin D.B., Hoffman B.M. (1993). Comprehensive explanation of the anomalous epr-spectra of wild-type and mutant cytochrome-C peroxidase compound-Es. Biochemistry.

[28] Casadei C.M., Gumiero A., Metcalfe C.L., Murphy E.J., Basran J., Concilio M.G. (2014). Neutron cryo-crystallography captures the protonation state of ferryl heme in a peroxidase. Science.

[29] Pond A.E., Bruce G.S., English A.M., Sono M., Dawson J.H. (1998). Spectroscopic study of the compound ES and the oxoferryl compound II states of cytochrome c peroxidase: comparison with the compound II of horseradish peroxidase. Inorg. Chim. Acta.

[30] Kwon H., Basran J., Casadei C.M., Fielding A.J., Schrader T.E., Ostermann A. (2016). Direct visualization of a Fe(IV)-OH intermediate in a heme enzyme. Nat. Commun..

[31] Ivancich A., Dorlet P., Goodin D.B., Un S. (2001). Multifrequency high-field EPR study of the tryptophanyl and tyrosyl radical intermediates in wild-type and the W191G mutant of cytochrome c peroxidase. J. Am. Chem. Soc..

[32] Jeschke G. (2005). EPR techniques for studying radical enzymes. Biochim. Biophys. Acta.

[33] Kolberg M., Bleifuss G., Sjoberg B.M., Graslund A., Lubitz W., Lendzian F. (2002). Generation and electron paramagnetic resonance spin trapping detection of thiyl radicals in model proteins and in the R1 subunit of Escherichia coli ribonucleotide reductase. Arch. Biochem. Biophys..

[34] Kolberg M., Bleifuss G., Graslund A., Sjoberg B.M., Lubitz W., Lendzian F. (2002). Protein thiyl radicals directly observed by EPR spectroscopy. Arch. Biochem. Biophys..

[35] Bleifuss G., Kolberg M., Potsch S., Hofbauer W., Bittl R., Lubitz W. (2001). Tryptophan and tyrosine radicals in ribonucleotide reductase: a comparative high-field EPR study at 94 GHz. Biochemistry.

[36] Gerfen G.J., Bellew B.F., Un S., Bollinger J.M., Stubbe J., Griffin R.G. (1993). High-frequency (139.5 GHz) EPR spectroscopy of the tyrosyl radical in Escherichia coli ribonucleotide reductase. J. Am. Chem. Soc..

[37] Fielding A.J., Brodhun F., Koch C., Pievo R., Denysenkov V., Feussner I. (2011). Multifrequency electron paramagnetic resonance characterization of PpoA, a CYP450 fusion protein that catalyzes fatty acid dioxygenation. J. Am. Chem. Soc..

[38] Fielding A.J., Singh R., Boscolo B., Loewen P.C., Ghibaudi E.M., Ivancich A. (2008). Intramolecular electron transfer *versus* substrate oxidation in lactoperoxidase: investigation of radical intermediates by stopped-flow absorption spectrophotometry and (9-285 GHz) electron paramagnetic resonance spectroscopy. Biochemistry.

[39] Macdonald I.K., Badyal S.K., Ghamsari L., Moody P.C., Raven E.L. (2006). Interaction of ascorbate peroxidase with substrates: a mechanistic and structural analysis. Biochemistry.

[40] Murphy E.J., Metcalfe C.L., Basran J., Moody P.C., Raven E.L. (2008). Engineering the substrate specificity and reactivity of a heme protein: creation of an ascorbate binding site in cytochrome c peroxidase. Biochemistry.

[41] Pearl N.M., Jacobson T., Meyen C., Clementz A.G., Ok E.Y., Choi E. (2008). Effect of single-site charge-reversal mutations on the catalytic properties of yeast cytochrome c peroxidase: evidence for a single, catalytically active, cytochrome c binding domain. Biochemistry.

[42] Leesch V.W., Bujons J., Mauk A.G., Hoffman B.M. (2000). Cytochrome c peroxidase cytochrome c complex: locating the second binding domain on cytochrome c peroxidase with site-directed mutagenesis. Biochemistry.

[43] Jasion V.S., Poulos T.L. (2012). Leishmania major peroxidase is a cytochrome c peroxidase. Biochemistry.

[44] Moody P.C.E., Raven E.L. (2018). The nature and reactivity of ferryl heme in compounds I and II. Acc. Chem. Res..

[45] Murphy E.J., Metcalfe C.L., Nnamchi C., Moody P.C., Raven E.L. (2012). Crystal structure of guaiacol and phenol bound to a heme peroxidase. FEBS J..

[46] Clark D., Albrecht M., Arevalo J. (1994). Ascorbate variations and dehydroascorbate reductase activity in trypanosoma cruzi epimastigotes and trypomastigotes. Mol. Biochem. Parasitol..

[47] Levine M., Conry-Cantilena C., Wang Y., Welch R.W., Washko P.W., Dhariwal K.R. (1996). Vitamin C pharmacokinetics in healthy volunteers: evidence for a recommended dietary allowance. Proc. Natl. Acad. Sci. U. S. A..

[48] Nelson D.P., Kiesow L.A. (1972). Enthalpy of decomposition of hydrogen-peroxide by catalase at 25 degrees C (with molar extinction coefficients of H_2_O_2_ solutions in Uv). Anal. Biochem..

[49] McCoy A.J., Grosse-Kunstleve R.W., Adams P.D., Winn M.D., Storoni L.C., Read R.J. (2007). Phaser crystallographic software. J. Appl. Crystallogr..

[50] Vagin A.A., Steiner R.A., Lebedev A.A., Potterton L., McNicholas S., Long F. (2004). REFMAC5 dictionary: organization of prior chemical knowledge and guidelines for its use. Acta Crystallogr. D Biol. Crystallogr..

[51] Emsley P., Lohkamp B., Scott W.G., Cowtan K. (2010). Features and development of coot. Acta Crystallogr. Sect. D.

[52] Altschul S.F., Gish W., Miller W., Myers E.W., Lipman D.J. (1990). Basic local alignment search tool. J. Mol. Biol..

[53] Aslett M., Aurrecoechea C., Berriman M., Brestelli J., Brunk B.P., Carrington M. (2010). TriTrypDB: a functional genomic resource for the trypanosomatidae. Nucl. Acids Res..

[54] Edgar R.C. (2004). Muscle: a multiple sequence alignment method with reduced time and space complexity. BMC Bioinform..

[55] Schneider T.D., Stephens R.M. (1990). Sequence logos - a new way to display consensus sequences. Nucl. Acids Res..

[56] Crooks G.E., Hon G., Chandonia J.M., Brenner S.E. (2004). WebLogo: a sequence logo generator. Genome Res..

[57] Jorgensen W.L., Chandrasekhar J., Madura J.D., Impey R.W., Klein M.L. (1983). Comparison of simple potential functions for simulating liquid water. J. Chem. Phys..

[58] Maier J.A., Martinez C., Kasavajhala K., Wickstrom L., Hauser K.E., Simmerling C. (2015). ff14SB: improving the accuracy of protein side chain and backbone parameters from ff99SB. J. Chem. Theor. Comput..

[59] Capece L., Lewis-Ballester A., Marti M.A., Estrin D.A., Yeh S.R. (2011). Molecular basis for the substrate stereoselectivity in tryptophan dioxygenase. Biochemistry.

[60] Case D.A., Ben-Shalom I.Y., Brozell S.R., Cerutti D.S., Cheatham T.E., I (2018).

[61] Rastelli G., Del Rio A., Degliesposti G., Sgobba M. (2010). Fast and accurate predictions of binding free energies using MM-PBSA and MM-GBSA. J. Comput. Chem..

[62] Humphrey W., Dalke A., Schulten K. (1996). Vmd: visual molecular dynamics. J. Mol. Graph.

[63] Jurrus E., Engel D., Star K., Monson K., Brandi J., Felberg L.E. (2018). Improvements to the APBS biomolecular solvation software suite. Protein Sci..

[64] Dalton D.A., Everse J., Everse K.E., Grisham M.B. (1991). Peroxidases in Chemistry and Biology.

