# Supplementary material for: Crystal structure of Trypanosoma cruzi heme peroxidase and characterization of its substrate specificity and compound I intermediate
Source: J Biol Chem. 2022 Jun 27;298(8):102204. doi: 10.1016/j.jbc.2022.102204 (PMC9358470; doi:10.1016/j.jbc.2022.102204)
Supplement: Figure S1 [file mmc1.pdf]

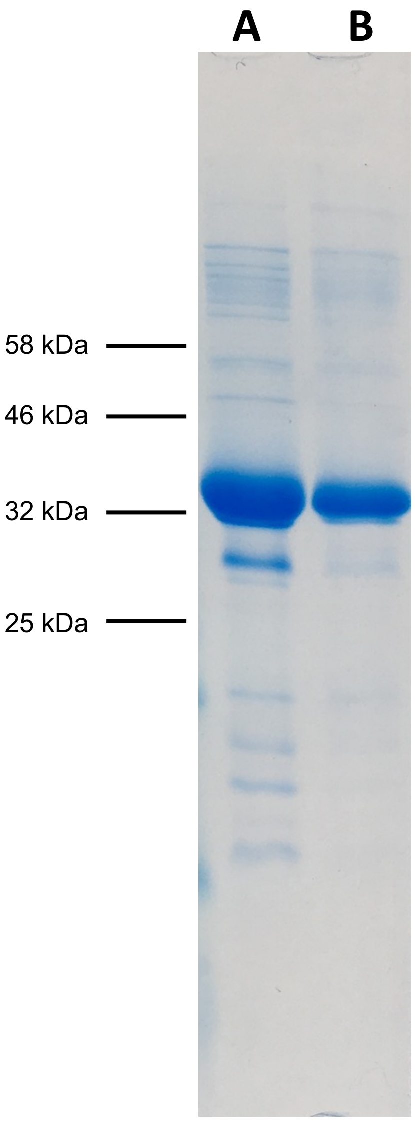

Figure S1 – SDS-PAGE of the combined early (A) and late (B) fractions from the nickel affinity chromatography step of the wildtype *Tc*APx-C*c*P. Molecular weight markers indicated on the left are derived from the ladder: New England Biolabs P7712S.
