# Supplementary material for: Crystal structure of Trypanosoma cruzi heme peroxidase and characterization of its substrate specificity and compound I intermediate
Source: J Biol Chem. 2022 Jun 27;298(8):102204. doi: 10.1016/j.jbc.2022.102204 (PMC9358470; doi:10.1016/j.jbc.2022.102204)
Supplement: Figure S2 [file mmc2.pdf]

Figure S2 Freeman *et al*

A

```
CcP           ------------------------------------------TTPLVHVASVEKGRSYED      18
TcAPXCcP      MAFCFGSFFSKYASSKSGSQARYRFLHSSAKIAAGATGALLLGGATVALCYFPSGRKV--      58
sAPX          -----------------------------------------------------SGKSYPT      7
                                                                   :*::

CcP           FQKVYNAIALKLR--EDDEYDNYIGYGPVLVRLAWHTSGTWDKHDNTGGSYGGTYRFKK-      75
TcAPXCcP      -TEAPPFDVNSLRRDIEEILSEDMSKGPLFVRLAWHEAGSWDCRKKDGSPNSASMRFHP-      116
sAPX          VSADYQKAVEKAKKKLRGFIAEKRC-APLMLRLAWHSAGTFDKGTKTGGPFGTIK--HPA      64
                      : : :         :   :*:::***** :*::*    :*:   :    :

CcP           EFNDPSNAGLQNGFKFLEPIHKEFPWISSGDLFSLGGVTAVQEMQGPKIPWRCGRVDTPE      135
TcAPXCcP      ECSYAGNKGLDKGRNALESLKKKYPKISYADLWSFAAVVSIEAMGGPEIPWRWGRVDAKD      176
sAPX          ELAHSANNGLDIAVRLLEPLKAEFPILSYADFYQLAGVVAVEVTGGPEVPFHPGREDKPE      124
              *    :* **: : : ** :: ::* :* :*:::::::*::::  **::*:: ** *  :

CcP           DTT-PDNGRLPDADKDADYVRTFFQR-LNMNDREVVALMGAHALGKTHLKNSGYEGPWGA      193
TcAPXCcP      GSVCGPDGRLPDASRMQDHVRDVFSR-LGFNDEETVALIGAHTCGECHLENTGYVGPWTH      235
sAPX          PP---PEGRLPDATKGSDHLRDVFGKAMGLTDQDIVALSGGHTIGAAHKERSGFEGPWTS      181
                    :****** :  *::* :* : ::::*:: *** *:*: *  * :::*: ***

CcP           ANNVFTNEFYLNLLNEDWKLEKNDANNEQWD-SKSGYMMLPTDYSLIQDPKYLSIVKEYA      252
TcAPXCcP      DKYGFDNSFFTELFGNEWMLNPNVKKMQFMDKTTNRLMMLPADVSILLDDKYRSIAKKYA      295
sAPX          NPLIFDNSYFTELLSGE----------------KEGLLQLPSDKALLSDPVFRPLVDKYA      225
                  * *::: :*:: :                ::  : **:* ::: *  :  ::::**

CcP           NDQDKFFKDFSKAFEKLLENGITFPKDAPSPFIFKTLEEQGL     294
TcAPXCcP      DDNDYFCNAFSKAYQKLLEVGTTDLKSLPAESK---------     328
sAPX          ADEDAFFADYAEAHQKLSELGFADA-----------------     250
               *:* *   :::*::** * * :
```

B

```
TcAPXCcP          1 MAFCFGSFFSKYASSKSGSQARYRFLHSSAKIAAGATGALLLGGATVALC     50
                                   .||:..|.:.|.:...:....:||:.:..|:..:
LmP               1 ---------------MSGTSRRAKGLFTGIAVGTFVSGAMFVSCASARV-     34

TcAPXCcP         51 YFPSGRKVTEAPPFDVNSLRRDIEEILSEDMSKGPLFVRLAWHEAGSWDC    100
                             |.||||:.:||.|||:::||.:..||..:|||||||.|:||
LmP              35 ---------EEPPFDIRALRADIEDMISEKLELGPSLIRLAWHEAASYDC     75

TcAPXCcP        101 RKKDGSPNSASMRFHPECSYAGNKGLDKGRNALESLKKKYPKISYADLWS    150
                    .|||||||||||||.|||.||||||||..|.|||:||||||:|||||||.
LmP              76 FKKDGSPNSASMRFKPECLYAGNKGLDIPRKALETLKKKYPQISYADLWV    125

TcAPXCcP        151 FAAVVSIEAMGGPEIPWRWGRVDAKDGSVCGPDGRLPDASRMQDHVRDVF    200
                    .||.|:||.||||.||:.||||||||||||||||||||.|:.|.|||:||
LmP             126 LAAYVAIEYMGGPTIPFCWGRVDAKDGSVCGPDGRLPDGSKTQSHVREVF    175

TcAPXCcP        201 SRLGFNDEETVALIGAHTCGECHLENTGYVGPWTHDKYGFDNSFFTELFG    250
                    .||||||:|||||||||||||||:|.:||.|||||||.||||||||:|..
LmP             176 RRLGFNDQETVALIGAHTCGECHIEFSGYHGPWTHDKNGFDNSFFTQLLD    225

TcAPXCcP        251 NEWMLNPNVKKMQFMDKTTNRLMMLPADVSILLDDKYRSIAKKYADDNDY    300
                    .:|:|||.|.:||.||:.|.:|||||:||.:|||..||...:.||.|||.
LmP             226 EDWVLNPKVEQMQLMDRATTKLMMLPSDVCLLLDPSYRKYVELYAKDNDR    275

TcAPXCcP        301 FCNAFSKAYQKLLEVGTTDLKSLPAESK    328
                    |...|:.|::||.|:||.:|...||...
LmP             276 FNKDFANAFKKLTELGTRNLHKAPASES    303
```

Fig. S2. (*A*) Multiple sequence alignments of *Tc*APx-C*c*P, soybean APX and yeast C*c*P. *Tc*APx-C*c*P has 32% and 34% identity with APX and C*c*P, respectively. Active site residues as indicated in Fig. 1 and Fig. 2 are in **bold;** residues in the region of the ascorbate binding site (R172 in APX, N226 in *Tc*APx-C*c*P and N184 in C*c*P, Fig. 5) are highlighted in red. Residues important for cytochrome *c* binding (Asp34, Glu35 in CcP) are in blue; residues Glu79 and Asp80 (also in blue) align well with Asp34 and Glu35 in the structure (not shown). Residues that conserved across are conserved across 2 proteins are marked with **:** underneath the sequence; those that are conserved across all 3 proteins are marked with ***.** (*B*) Sequence alignment of *Tc*APx-C*c*P and *Lm*P (57% sequence identity). Active site residues as indicated in Fig. 1 are in bold; F201 in LmP (equivalent to N226 in *Tc*APx-C*c*P) is also in bold.
