# Supplementary material for: Crystal structure of Trypanosoma cruzi heme peroxidase and characterization of its substrate specificity and compound I intermediate
Source: J Biol Chem. 2022 Jun 27;298(8):102204. doi: 10.1016/j.jbc.2022.102204 (PMC9358470; doi:10.1016/j.jbc.2022.102204)
Supplement: Figure S3 [file mmc3.pdf]

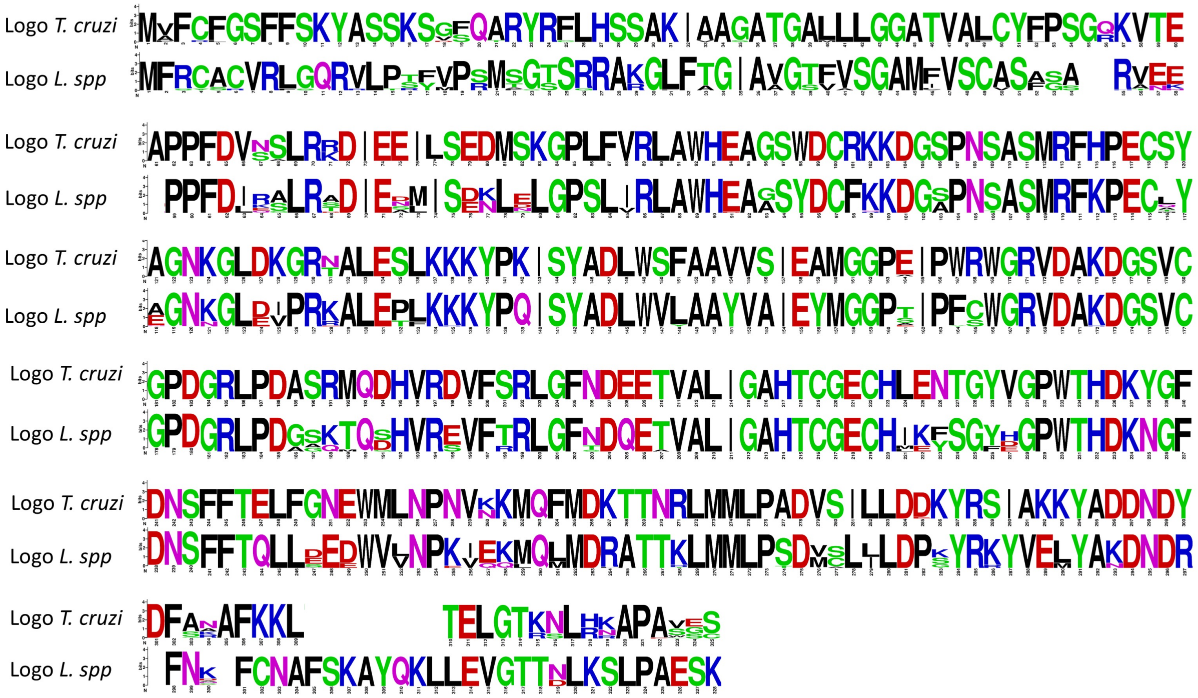

Fig. S3. Sequence logos obtained from the *Tc*APx-CcPs sequence alignments of all well-annotated *T. cruzi* genomes available, as well for the homologous gene from every *Leishmania spp.* genome available.
