# Supplementary material for: Crystal structure of Trypanosoma cruzi heme peroxidase and characterization of its substrate specificity and compound I intermediate
Source: J Biol Chem. 2022 Jun 27;298(8):102204. doi: 10.1016/j.jbc.2022.102204 (PMC9358470; doi:10.1016/j.jbc.2022.102204)
Supplement: Figure S4 [file mmc4.pdf]

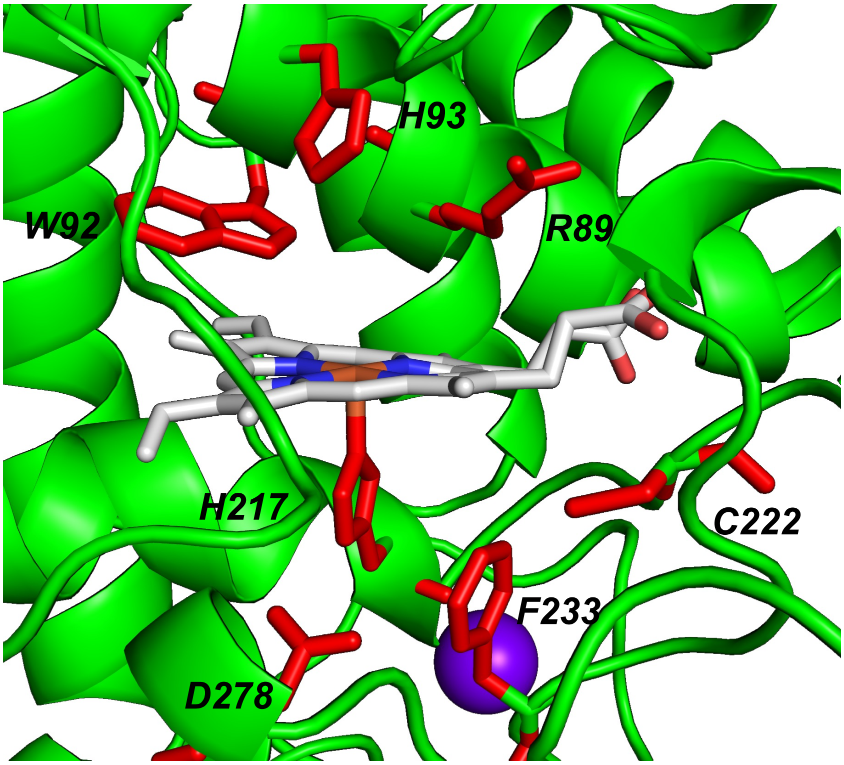

Figure S4. The structure of the heme in the *Tc*APx-CcP W233F variant. The mutated residue F233 is shown as red sticks along with other important residues in the heme pocket. There is an active site sodium atom, which is shown as a purple sphere.
