# Supplementary material for: Crystal structure of Trypanosoma cruzi heme peroxidase and characterization of its substrate specificity and compound I intermediate
Source: J Biol Chem. 2022 Jun 27;298(8):102204. doi: 10.1016/j.jbc.2022.102204 (PMC9358470; doi:10.1016/j.jbc.2022.102204)
Supplement: Figure S5 [file mmc5.pdf]

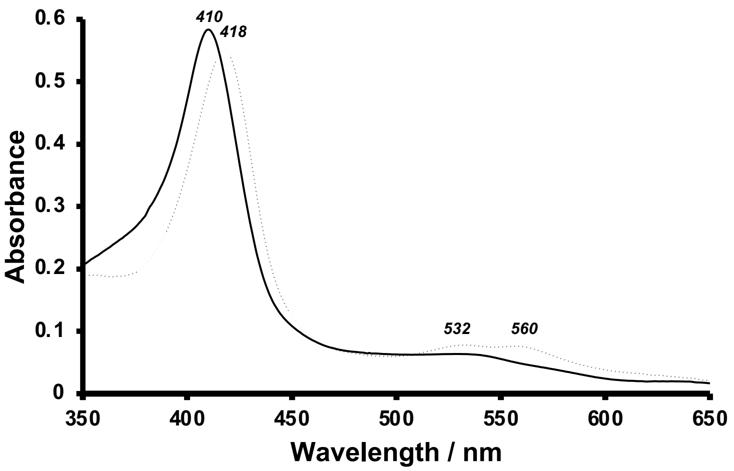

Figure S5. Reaction of C222A (5 µM, solid line) obtained after reaction of equimolar H_2O_2 (dotted line) in a stopped-flow spectrophotometer at 10°C in 50 mM potassium phosphate buffer pH 7.4.
